# Self-medication practices against COVID-19 infection and awareness among residents of Mogadishu, Somalia: A cross-sectional analysis

**DOI:** 10.1371/journal.pone.0284854

**Published:** 2023-06-28

**Authors:** Ayan Aden Moussa, Fardosa Dahir Omar, Osman Abubakar Fiidow, Fadumo Hassan Ali, Sadiq Mohammed Babatunde

**Affiliations:** 1 Institute for Medical Research, SIMAD University, Mogadishu, Somalia; 2 Faculty of Medicine and Health Science, SIMAD University, Mogadishu, Somalia; 3 Faculty of Veterinary Medicine, Universiti Putra Malaysia, Serdang, Selangor, Malaysia; World Health Organization, SOUTH SUDAN

## Abstract

The novel coronavirus disease (COVID-19) pandemic has affected several countries worldwide, resulting in a considerable strain on healthcare systems and increased trend of self-medication practices. This study aims to evaluate the awareness of COVID-19 and the prevalence of self-medication during the pandemic among residents in Mogadishu, Somalia. A cross-sectional study was conducted using a structured and pretested questionnaire between May 2020 and January 2021. Participants from various disciplines were randomly recruited within the study location and interviewed about their self-medication practices during the pandemic. Descriptive statistics were used to summarise the respondents’ information and responses to the questionnaire items. Associations between participants’ demographic characteristics and specific items relating to self-medication practices were analysed using the Chi-square test. A total of 350 residents participated in the study. Approximately 63% of the participants reported having practised COVID-19 related self-medication with the main reasons being pharmacists’ advice (21.4%) and having an old prescription (13.1%), whereas 37.1% did not report their reasons for self-medication. Most participants (60.4%) engaged in self-medication despite not having any symptoms and 62.9% had taken antibiotics in the last three months. Most participants were aware that no medication has been approved for COVID-19 (81.1%), the negative effects of self-medication (66.6%), and the transmission routes of the virus. Meanwhile, more than 40% of the participants have not worn a mask while outside their homes, and do not follow the international COVID-19 guidelines. The most prevalent drug used by participants for self-medication against COVID-19 was paracetamol (81.1%) and antibiotics (78%). The factors associated with awareness of COVID-19 and self-medication practices included age, gender, educational qualification, and occupation. This study revealed considerable high self-medication practices among Mogadishu residents, thus highlighting the need to promote awareness regarding the adverse effects of self-medication and sanitisation guidelines in addressing COVID-19 at the community level.

## Introduction

The fear and apprehension following the outbreak of COVID-19 and its subsequent global spread to most continents of the world are palpable in Africa. Factors such as high population density, poorly funded public health sector, and lack of testing facilities in most African countries led to the projections by global public health experts that the pandemic could be catastrophic in the region [1]. Other critical factors that heightened the fear were inadequate health infrastructure, and a lack of skilled and well-trained human capital to facilitate the collection, processing, and testing of patient samples [2].

From the onset of the pandemic to date, no evidence-based treatments are available for COVID-19 despite the devastation and global spread of the virus. Hence, several countries have resorted to granting authorisation for the use of monoclonal antibody treatments for non-hospitalised adults and children over 12 years old with mild to moderate COVID-19 symptoms [3]. These treatment options are a combination of either casirivimab and imdevimab, or bamlanivimab and etesevimab, recommended especially for those at risk of developing severe COVID-19 or being hospitalised [4,5]. Other less popular options are non-steroidal anti-inflammatory drugs and hydroxychloroquine [4]. Despite the seeming success of these treatments against COVID-19, the World Health Organisation (WHO) declared that clinical trials on these drugs were discontinued due to increased risk of ventricular arrhythmias and death among hospitalised patients [6]. Unfortunately, many African countries have already approved the use of these drugs to treat COVID-19 at the national level as against the WHO directives [6]. The use of these substances without appropriate medical advice as in the case of most African countries is considered self-medication [7].

The WHO defined self-medication as the use of medicinal products, including over the counter (OTC) or non-prescription drugs as well as prescription-only medicines to treat self-diagnosed symptoms or illnesses without obtaining advice from a physician, pharmacist or other healthcare personnel in the diagnosis [8]. It is also the intermittent or continuous use of medication previously prescribed by a physician for chronic or recurring symptoms or diseases. Several studies have reported an increase in the prevalence of self-medication among various populations during the COVID-19 pandemic, including 83% of medical students in Pakistan [9], and 36.3% of healthcare professionals in Nigeria [10], and 34.2% among multidisciplinary participants in Togo [7]. These events have been linked to the widespread psychosis and anxiety induced among populations in sub-Saharan Africa due to the COVID-19 epidemic [11]. Perceivably, the high mortality rates reported in Spain and Italy, as well as the lack of technical resources to address the disease in sub-Saharan Africa [12]. Given the aforementioned situations and the variety of information circulating on social media, numerous substances without the minimum requirements of efficacy and tolerance are taken by individuals either for COVID-19 treatment or prophylaxis [12].

Like most African countries, Somalis practice self-medication due to a lack of regulation and the ease at which drugs can be bought OTC without prescription [13]. This improper usage of drugs for COVID-19 treatment has the potential to complicate case management and induce toxic effects in patients and increase the risk and emergence of drug resistance, which could increase the rate of hospitalisation and severe infection with drug-resistant pathogens [10]. Somalia has been in turmoil for decades and is among the countries with the weakest health systems in Africa. The country is struggling with a new wave of COVID-19 infections with 14,632 confirmed cases and 767 death as of 25th May 2021, and this may likely surge leading to unrecorded and unrecognised deaths [14]. Besides, the reported statistics may not reflect the reality on the ground given that testing for COVID-19 cases has been comparatively limited to major cities, such as Mogadishu, Kismayo, and Marka. Meanwhile, all regions currently facing security challenges are been denied access [14]. Based on the lack of a recognised treatment for COVID-19 and its constant progression, self-medication practices among Somalis need to be evaluated. Given the significance of these issues with the pandemic, this study was conducted to assess the awareness level of COVID-19 and self-medication practices against SARS-CoV-2 viral infection among Somalis.

## Methodology

### Study design, sample size determination and sampling

This is a cross-sectional study conducted among Mogadishu residents in Somalia from May 2020 to January 2021. Mogadishu was selected for this study as the capital of Somalia and the first case of COVID-19 was recorded in the city. Moreover, it is a commercial state with the highest population in the country [15].

A pre-validated questionnaire was administered to the participants via an online platform (google form) and paper format. Participants were recruited from three main sectors: Education (academicians, lecturers, and teachers), Government parastatals (Civil service, government hospitals, and public utilities), and informal/private sectors (farmers, market workers, and business owners). These sectors were targeted given the increased risk of contracting COVID-19 in such workplaces [16]. Inclusion criteria were residents from Mogadishu, aged 18 years and above, and currently working or aligning with any of the aforementioned sectors. Additionally, oral consent and willingness to participate in the survey were necessary before recruitment.

The required sample size was calculated using a single proportion formula with a 95% confidence level. We hypothesized that 34.2% of the population will engage in self-medication practice against COVID-19, with a precision error of 5%. This expected prevalence of self-medication practices was chosen based on a study conducted in Togo [7]. The study was used as a reference since it was a cross-sectional design involving participants from sub-Saharan Africa, involving a cross-sectional design. Hence, the estimated minimum sample size was 350. Upon adjusting for a non-response rate of 10%, the sample size was increased to 400 potential participants.

Random sampling was performed based on the estimated number of people at risk of contracting COVID-19 in the various districts of Mogadishu. The criteria were based on patient characteristics and detailed comorbidities at the community healthcare centres as described by Booth et al. [16]. The number of households in the districts was documented, followed by randomly selecting specific households using a computer-generated dataset. Thereafter, a single individual was selected from the households to participate in the study. A total of 400 individuals from various disciplines were selected and their preferred means of responding to the questionnaire were documented. Although participants were recruited at random, only those within the study location and considered to be at risk of contracting and suffering COVID-19 infection (excluding children below 18 years) were included.

### Development of the survey instrument

The structured questionnaire was developed in English and no translation process was necessary since the participants use English as a medium of communication. The questionnaire was synthesised based on the review of recent studies relating to self-medication practices among various populations [7,17,18]. Nevertheless, self-medication practices suggested to be unique among the Somalis were also considered while developing the questionnaire. All the authors of this manuscript participated in developing the instrument.

The questionnaire comprised four sections: sociodemographic characteristics, reasons and symptoms for self-medication against COVID-19, awareness and practices relating to COVID-19 and specific self-medication used during the pandemic. The first section consisted of participants’ personal information such as gender, highest educational level, occupation, age, and the number of family or household members. The section entailed questions to assess the prevalence of self-medication practices, reasons for self-medication, conditions warranting self-medication in the last 90 days, history of antibiotic therapy, and rationale for such practices. Participants were provided specific options or dichotomous responses (True or False). In the third section, the questions were designed to assess participants’ awareness regarding currently approved medication for COVID-19, the impact of self-medication, transmission routes of the virus, and preventive measures. Furthermore, participants were asked pertinent questions concerning previous exposure, status, and symptoms relating to COVID-19. The last section focused on prophylactic self-medications such as the use of vitamin C, hydroxychloroquine, paracetamol, antibiotics, and traditional medicine. Traditional medicine included if either any plants or herbal products have been used by participants.

### Measurements

Self-medication practice was considered the dependent or outcome variable in this study. The variable was measured using a dichotomous item (True/False). Awareness and self-medication practices relating to COVID-19 were also measured using Yes or No responses, however, the items were scored based on participants’ responses (score 1 for a correct answer and 0 for a wrong answer). Thus, the overall score was computed as a continuous outcome.

### Questionnaire administration

Upon ascertaining the participants’ eligibility, the standardised questionnaire was pre-tested on 20 selected individuals who were not deployed for the actual survey. This pilot testing was conducted to ensure that the main participants comprehend the items in the questionnaire, and to determine the internal consistency and reliability of the instrument. Data from the pilot test were analysed and employed in validating the questionnaire. The questionnaire was re-evaluated by a researcher who was not involved in the instrument design. Resultantly, the instrument was judged to be reliable and no further adjustment was undertaken. Thereafter, the questionnaire was administered to the participants via two methods: online google and paper format. The questionnaire was administered to each according to the preferred method selected during recruitment. The link for the online google form was sent directly using to participants’ email repository. Meanwhile, two enumerators administered the questionnaire using the paper format.

### Statistical analysis

All the statistical analyses were performed using the Statistical Package for Social Science (SPSS) version 23.0. A reliability test was performed using factor analysis and items were considered internally consistent based on the Cronbach’s alpha value > 0.6. Descriptive statistics were used to summarise the data. A normality test was conducted using the level of kurtosis and skewness. Mean and standard deviation was applied to present the normally-distributed continuous data, whereas categorical data were summarized using frequencies and percentages. Awareness and self-medication practices relating to COVID-19 were measured using Yes or No responses, however, the items were scored based on participants’ responses (score 1 for a correct answer and 0 for a wrong answer). Thus, the overall score was computed as means and standard deviations. Associations between participants’ demographic characteristics and specific items relating to self-medication practices were analysed using the Chi-square test. A p-value < 0.05 was considered for significant associations.

### Ethical approval and participants’ consent

Participants were duly informed about the research objectives, as well as the confidentiality of all data and information provided. Hence, they were shown a copy of the questionnaire indicating that no personal data were required that could reveal participants’ identities. They were briefed that the survey poses no potential risk. Those willing to participate were given a consent form to fill and sign before the questionnaire was administered.

## Results

### Participants’ socio-demographic characteristics

Out of the 420 persons that received the questionnaire, 350 responded which resulted in a response rate of 83.3%. Among the participants, 60.6% of them were males and 57.4% belonged to the age group of 26–35 years old (Table 1). A higher proportion had tertiary educational qualifications (58.6%), which was reflected in the prevalent occupation as most participants belong to the academic field (54.0%). The mean (± SD) number of family members in the participants’ households was 5.6 (± 1.2).

**Table 1:** Participants’ socio-demographic characteristics.

Variables	Frequency	Percentage
**Gender**		
**Male**	212	60.6
**Female**	138	39.4
**Educational qualification**		
**Primary**	26	7.4
**Secondary**	42	12.0
**Tertiary**	205	58.6
**Non-formal**	55	15.7
**Others**		
**Occupation**		
**Academic**	189	54.0
**Civil servants**	43	12.3
**Business**	53	15.1
**Farmer**		
**Others**	13	3.7
**Age (in years)**		
**18–25**	107	30.5
**26–35**	201	57.3
**36–50**	35	10.0
**Above 50**	8	2.3
**Total number of families living together (mean ± SD)**	5.6 ± 2.54	

SD = standard deviation.

### Prevalence and symptoms of self-medication practices

Table 2 reports the overall self-medication prevalence and reasons stated for self-medicating. The overall prevalence of the use of drugs to treat or prevent COVID-19 without any medical advice was 62.9% (n = 220). A higher proportion of participants stated pharmacists’ advice (21.4%) prompted them to engage in self-medication, followed by having a previous prescription (13.1%) and distance from their homes to hospitals (10.9%). However, 37.1% of them did not state their reasons for self-medicating.

**Table 2:** Prevalence of self-medication against COVID-19, reasons, and symptoms for self-medication among residents from Mogadishu in Somalia (n = 350).

Statements/items	Frequency	Percentage
**Have you taken self-medication in the last 3 months**		
**Yes**	220	62.9
**No**	130	37.1
**Reasons for self-medication**		
**Hospital far from home**	38	10.9
**High cost**	32	9.1
**Pharmacist advice**	75	21.4
**I have an old prescription**	46	13.1
**Others**	29	8.3
**N/A**	130	37.1
**Conditions warranting self-medication in the last 3 months**		
**Fever**	53	15.1
**Body aches**	17	4.9
**Headache**	49	14.0
**Cough**	28	9.0
**Loss of taste or smell**	5	1.4
**Difficulty breathing**	31	8.9
**Sore throat**	28	8.0
**Prevention for COVID-19**	5	1.4
**No symptoms**	139	39.7
**Have you ever taken antibiotics in the last 3 months when ill?**		
**Yes**	220	62.9
**No**	130	37.1
**Your selection of antibiotics was based on**		
**Pharmacist’s advice**	73	20.9
**Previous prescription**	116	33.1
**Family members**	34	9.7
**Friends’ opinions**	29	8.2
**Self-experience**	19	5.4
**Advertisement**	0	0
**N/A**	77	22.0

N/A = None of the above.

Regarding the conditions that necessitated self-medication, an alarming 39.7% of participants took medications without having any symptoms. Meanwhile, common symptoms for which the respondents took medications were fever or chills (15.1%,) headache (14%), while the least reported as a loss of smell or taste (1.4%). A higher proportion of participants (62.9%) have taken antibiotics in the last 90 days for therapeutic purposes. Similarly, pharmacists’ advice (20.9%) and previous prescriptions (33.1%) were the dominant reasons for self-antibiotic medication.

### Awareness and self-medication practices relating to COVID-19 infection

Participants demonstrated satisfactory awareness levels as 81.1% stated that no medication has been approved for the treatment of COVID-19 (Table 3). More than 65% of participants were aware that self-medication against COVID-19 may affect their health adversely and that the virus is airborne. Furthermore, 92% posited that the virus spreads via the respiratory droplets of infected individuals.

**Table 3:** Awareness and self-medication practices relating to COVID-19 infection.

S/N	Statements/items	Frequency	Percentage
**1**	There is still no approved medication for COVID-19		
	True	65	18.9
	False	284	81.1
**2**	Self-medication can affect your health adversely		
	True	233	66.6
	False	117	33.4
**3**	The COVID-19 virus is airborne		
	True	297	84.9
	False	53	15.1
**4**	The COVID-19 virus spreads via respiratory droplets of infected individuals		
	True	322	92.0
	False	28	8.0
**5**	Have you worn a mask when leaving home in recent days?		
	Yes	181	51.7
	No	69	48.3
**6**	Are you following the WHO guidelines?		
	Yes	209	59.7
	No	141	40.3
**7**	How often do you wash your hands?		
	0		
	1–5 times	82	23.4
	6–8 times	89	25.4
	More than 8 times	179	51.2
**8**	Have you been to any crowded places in recent days?		
	Yes	238	68.0
	No	112	32.0
**9**	Have you been diagnosed with COVID-19		
	Yes	62	17.7
	No	288	82.3
**10**	If yes, were you hospitalised?		
	Yes	40	64.5
	No	22	35.5
**11**	Have you had close contact with someone who has laboratory-confirmed COVID-19		
	Yes	90	25.7
	No	260	45.7
**12**	If yes, did you self-isolate?		
	Yes	24	26.7
	No	66	73.3
**13**	Have you had any of the following signs in the last 7 days		
	Fever	49	14.0
	Body aches	32	9.1
	Headache	80	22.0
	Cough	23	6.5
	Loss of taste or smell	26	7.4
	Difficulty breathing	34	9.7
	Sore throat	19	5.4
	No	87	24.8

Table 3 depicts participants’ responses regarding preventive measures and practices against exposure to COVID-19. In recent days, only 51.7% of participants had worn a mask before leaving their homes while 68.0% have attended crowded events. Approximately 41% claimed not to be adhering to international guidelines although 51% stated they wash their hands more than eight times daily.

In this study, only 17.7% of participants have been previously diagnosed with COVID-19. Of the positive cases, 64.5% resulted in hospitalisation. While 25.7% had close contact with confirmed COVID-19 cases, most of them did not self-isolate (73.3%). The prevalent symptom manifested by participants in the last week before participating in this study was headache (22.0%) and fever (14.0%). Table 4 Overall, the most frequently used drug among the respondents for self-medication was paracetamol (81.1%), followed by antibiotics (78%), Vitamin C (56%), and the least used was hydroxychloroquine (31.4%). Most participants (93.7%) were not aware of the specific antibiotic used during self-medication.

**Table 4:** Specific self-medication used during the COVID-19 pandemic.

S/N	Statements/items	Frequency	Percentage
	**Drugs used for COVID-19**		
**1**	**Vitamin C**		
	Yes	199	56.0
	No	151	44.0
**2**	**Hydroxychloroquine**		
	Yes	110	31.4
	No	240	68.6
**3**	**Traditional medicine**		
	Yes	210	60.0
	No	140	40.0
**4**	**Paracetamol**		
	Yes	284	81.1
	No	66	18.9
**5**	**Antibiotic**		
	Yes	273	78.0
	No	66	22.0
**6**	**If yes, which antibiotic have you used**		
	Ampicillin	6	2.1
	Amoxicillin	1	0.4
	Augmentin	5	1.8
	Ceftriaxone	4	1.5
	Tetracycline	1	0.4
	Unknown	256	93.7

### Factors associated with participants’ awareness and self-medication practices

The factors associated with participants’ awareness and self-medication practices were age, educational level, occupation, and gender (Table 5). A higher mean score was obtained by participants aged 25–35 compared to other age groups. Those with tertiary educational qualifications had higher mean scores relative to participants with primary, high school, and non-formal educational education. Academicians demonstrated higher awareness and better practices relating to COVID-19 compared to those working in other fields (civil servants, business, and farming).

**Table 5:** Association between participants’ socio-demographic characteristics and awareness of COVID-19 and related preventive and self-medication practices.

Variables	No. of respondents	Mean (± SD) awareness and self-medication practices score	P-value
**Gender**			0.04
**Male**	212	4.80± 0.98^b^	
**Female**	138	5.72±1.48^a^	
**Educational qualification**			0.001
**Primary**	205	5.91±1.31^a^	
**Secondary**	42	4.95±1.20^b^	
**Tertiary**	26	4.46±1.02^b^	
**Non-formal**	22	4.41±0.93^b^	
**Others**	55	5.5±1.38^b^	
**Occupation**			0.001
**Academic**	189	5.90±1.38^b^	
**Civil servants**	43	5.27±1.07^a^	
**Business**	53	4.79±0.84^a^	
**Farmer**	13	4.69±1.25^a^	
**Others**	52	4.19±1.01^a^	
**Age (in years)**			0.01
18–25	107	4.78±1.30^a^	
26–35	201	5.84±1.26^b^	
36–49	34	4.50±1.21^a^	
**Above 50**	8	4.50±0.75^a^	

**Note**: Means with different superscripts are statistically different at P = 0.05.

## Discussion

This study investigated the practice of self-medication against COVID-19 among Somalis. Prior to this investigation, previous reports on self-medication focused on the general context among Somalis in Puntland and Somaliland [13]. Since the pandemic occurred in early 2020, this is the first study on self-medication practices and awareness regarding COVID-19 among Somalis.

Understanding the level of self-medication to treat COVID-19, the underlying reasons for such practices, and the associated factors will assist in preventing the potential detrimental effects. Self-medication practices tend to give individuals the impression that they are safe, hence they will lower their guards and not adhere to the recommended prevention and control measures against COVID-19. These events endanger both themselves and the public.

In this study, the overall prevalence of self-medication for COVID-19 treatment or prevention without prior medical or professional advice was 62.9%. This finding is comparably higher than the earlier reports in Nigeria at 41% [10], 34.2% in Togo [7] and 35.1% in Saudi Arabia [19], similar to estimates in Uganda at 57.0% [20], and lower compared to Bangladesh at 88.3% [21]. These differences might be attributed to variation in participants’ socio-demographic characteristics, the definition of self-medication, the severity of COVID-19, availability of drugs used for self-medicating, and study period and designs. For instance, this study used both online and paper formats in administering the survey, which is similar to the methods used by Dare et al. [20] in Uganda. Meanwhile, the recall periods, self-medication definitions, and investigation periods differed compared to the other studies.

Most respondents submitted that they self-medicated after exhibiting clinical signs synonymous with COVID-19 including chills and headache, whereas only a few respondents reported experiencing loss of smell or taste. These findings may imply that they engaged in self-medication after being aware of these signs being related to COVID-19. This on one hand shows that Somalis demonstrated the clinical manifestation of COVID-19 as reported elsewhere [7,10], but the decision to self-medicate was ill-advised.

This may not be unconnected with the fact that several respondents were wrongly advised to take these medications by pharmacists. This is true for this investigation as 21.4% reported that they self-medicated because of advice received from pharmacists. Similarly, about 13.1% followed their old prescriptions while 10.9% reported that the hospital was far from them to get a doctor’s prescription for the medication. Pharmacists have always played an important role in recommending on the counter medications to help patients deal with their health issues [18]. They often do so by asking some questions and then recommending certain medications [22]. The combination of location and accessibility means that most consumers have ready access to a pharmacy where attendants who most times are not pharmacists give advice and recommendations on demand. Meanwhile, the urge to use old prescriptions for illnesses in recent years is consistent with the behaviours of modern consumers or patients. They feel responsible to take a greater role in the maintenance of their health and are unwilling to submit to the inconvenience of visiting a doctor for what they feel they could manage by themselves [23].

Participants demonstrated satisfactory awareness levels in terms of no approved medications against COVID-19, adverse effects of self-medication, and the nature and transmission routes of the virus. These findings might be related to the fact that most participants had the tertiary educational qualification and were academicians. This result was confirmed in further analyses as those with the highest educational qualification recorded higher mean scores for awareness related items compared to those with lower educational qualifications. Similar findings have been reported in studies conducted in academic settings [24] and population-based [25] in which undergraduate students and the general population demonstrated good knowledge and awareness about COVID-19. Additionally, participants belonging to the middle age group (25–50 years) had higher mean awareness and knowledge scores compared to other age categories. This could also be related to the educational status of this age group as most are probably actively working in academic settings, and more exposed to online and current information.

A high proportion of participants in this study reflected poor practices toward COVID-19 preventive measures, such as not wearing masks, attending crowded events, not following international guidelines, and have engaged in self-medication. To elucidate these findings, females and older participants had higher mean scores for good practices relating to COVID-19 preventive measures compared to males and younger age groups, respectively. Meanwhile, no association was detected between education and participants’ practices. These results are consistent with the reports among Chinese [26], Iran [25] and South Korean populations [27] in which females demonstrated satisfactory practices toward the novel virus outbreak. This is particularly important in this study despite only 39.4% of the participating Somalis were females. Recent studies have also shown positive associations between increasing age and adhering to COVID-19 protocols [28]. As found in this study, given the good knowledge demonstrated by most participants, those belonging to the higher age group might be aware that COVID-19 causes more deleterious effects in the ageing population. Thus, there are more inclined to adhere to preventive measures such as avoiding crowding places, washing hands more frequently, and wearing masks compared to the younger population. This is supported by a recent longitudinal study conducted in Switzerland in which young adults were identified to comply less with COVID-19-related public health measures.

Nevertheless, there was no significant association between education and self-medication practices in this study. This observation is contrary to the general belief that abuse of drugs due to self-medication and violation of COVID-19 protocols is driven by ignorance and lack of awareness. For instance, Lee et al. [29] found that knowledge directly affected both practices such as social distancing and personal hygiene towards COVID-19 among South Korean residents. In other words, the level of education plays no part in determining the level of self-medication among respondents in the present study. Other factors such as efficacy belief might play a more vital role compared to educational level in influencing participants’ practices.

Among the drugs that were frequently consumed, most respondents take paracetamol (81.1%), followed by antibiotics (78.0%), and the least consumed hydroxychloroquine. Paracetamol can cause liver damage either at high doses or when combined with other substances that affect the liver. In addition, toxicity from paracetamol is one of the top causes of acute liver failure globally [30]. Ironically, 81.1% reported that no medication has been approved for COVID-19 but 18.9% believed these antibiotics are a good treatment for COVID-19. Misinformation from media outlets including social media platforms has contributed significantly to the devastation recorded since the first outbreak of COVID-19. The World Health Organisation reported that in the first three months of 2020, nearly 6000 were hospitalised and 800 people might have died due to COVID-19 misinformation [31].

Relevant authorities and other stakeholders in the fight against COVID-19 in Somalia need to create more awareness and counter these sources of misinforming the public. The hazards associated with imprudent use of antimicrobials need to be emphasised as it is now established as a major driving force in the development of antimicrobial resistance and the emergence of highly infectious resistant pathogens. Therefore, guidelines and regulations need to be enforced in safeguarding the distribution and sale of over the counter drugs in Somalia.

### Study limitations

This study is not without limitations. First, a cross-sectional study was conducted and data were collected at a single time point. Thus, only snapshot information was available and no casual inference could be drawn from this study. Second, the findings are not generalisable to other states in Somalia since only Mogadishu residents were sampled coupled with the relatively small sample size. Third, utilising a quantitative approach and structured questions also has its weaknesses. The technique is prone to response bias and detailed information cannot be gleaned. An in-depth interview and a larger sample size could be considered in future research.

### Conclusion

Conclusively, the use of different over-the-counter medications for the prevention and treatment of perceived COVID-19 by Somalis is more common among the residents of Mogadishu, which includes the educated class. Paracetamol and antibiotics were the most consumed drugs as a preventive measure against COVID-19 in Mogadishu, Somalia. The scourge of fake cures and unproven treatments promoted on social media across the globe has resulted in some of the most devastating outbreaks. Therefore, we recommend that all stakeholders, pharmacists, and the media be engaged to educate people on the best ways to manage suspected cases of COVID-19.

## Supporting information

S1 File (XLSX)
