# Supplementary material for: Self-medication practices against COVID-19 infection and awareness among residents of Mogadishu, Somalia: A cross-sectional analysis
Source: PLoS One. 2023 Jun 28;18(6):e0284854. doi: 10.1371/journal.pone.0284854 (PMC10306195; doi:10.1371/journal.pone.0284854)
Supplement: Attachment — Submitted filename: Response.docx-revised2023_PONE-D-22-11313.docx [file pone.0284854.s002.docx]

Author’s Response	30/12/2022

Thanks for taking your time to review our manuscript. Your comments and suggestions have really assisted the authors in improving the content and presentation of the manuscript. All your suggestions were thoughtful and helpful. We have attempted to respond to all the comments raised during the review.

Kindly see the response to each comment in the table presented below:

| **REVIEWER’S COMMENTS** | AUTHOR’S RESPONSE | PAGE |
| --- | --- | --- |
| The authors should number each line of their manuscript so that reviewers can exactly point out mistakes. | Number line has been included in the revised manuscript | All pages |
| **Abstract** |  |  |
| They should add some keywords such as awareness of COVID-19, self-medication practices, Mogadishu residents, and community-based cross-sectional study. | Keywords have been added:  Awareness, COVID-19, self-medication practices, Somalia, Prevalence | Page 2 |
| **INTRODUCTION** |  |  |
| The sentence "Some of the aforementioned therapies …the number of emergency room visits" should be cited obviously via Oxford – Author Data style instead of the phrase "(Reference)." - The authors should do the same for one similar error in the sentence "Like most African countries…over the counter without prescription". | All references have been cited using Vancouver style  Like most African countries, Somalis practice self-medication due to a lack of regulation and the ease at which drugs can be bought OTC without prescription [13]. | Page 3 |
| **Methodology** |  |  |
| Study design, sample size determination, and sampling - They should present which formulas they used to calculate the sample size. | All the information has been included in the revised manuscript.  The required sample size was calculated using a single proportion formula with a 95% confidence level. We hypothesized that 34.2% of the population will engage in self-medication practice against COVID-19, with a precision error of 5%. | Page 5 |
| They should clarify why they chose the study conducted in Togo by Sadio et al. (2021) rather than other relevant studies | The study was used as a reference since it was a cross-sectional design involving participants from sub-Saharan Africa, involving a cross-sectional design. | Page 5 |
| They need to display the selection criteria for the intended participants. | Although participants were recruited at random, only those within the study location and considered to be at risk of contracting and suffering COVID-19 infection (excluding children below 18 years) were included. | Page 5 |
| Development of instrument/ questionnaire - Three recent studies on self-medication practices cited, including Quinco-Lopez et al.,2021; Wegborn et al.,2021; Nasir et al.,2020, are not found in the References section. The authors did not add these studies to their References section. | All the studies have been cited in the references | Reference page (page 19) |
| Administration of question - They should mention the process for validating a questionnaire in more detail, such as which items needed to be re-edited after the pilot test, how these items were changed, and whether they had a committee of experts re-check the questionnaire after the pilot test. | Upon ascertaining the participants’ eligibility, the standardised questionnaire was pre-tested on 20 selected individuals who were not deployed for the actual survey. This pilot testing was conducted to ensure that the main participants comprehend the items in the questionnaire, and to determine the internal consistency and reliability of the instrument. Data from the pilot test were analysed and employed in validating the questionnaire. The questionnaire was re-evaluated by a researcher who was not involved in the instrument design. Resultantly, the instrument was judged to be reliable and no further adjustment was undertaken. | Page 6 |
| Ethical Approval and Participants' Consent - They should mention how the confidentiality of participants was guaranteed. | Participants were duly informed about the research objectives, as well as the confidentiality of all data and information provided. Hence, they were shown a copy of the questionnaire indicating that no personal data were required that could reveal participants’ identities. They were briefed that the survey poses no potential risk. Those willing to participate were given a consent form to fill and sign before the questionnaire was administered. | Page 7 |
| RESULTS |  |  |
| Prevalence and symptoms for self-medication practices - There was mismatched information on the percentage of respondents taking antibiotics between the Abstract and Results sections. The authors stated, "An alarming 62.1% of participants have taken antibiotics in the last three months" in Abstract, while they declared, | The values have been corrected.  A higher proportion (60.4%) engaged in self-medication despite not having any symptoms. An alarming 62.9% of participants have taken antibiotics in the last three months. | Page 1 |
| A higher proportion of participants (62.9%) have taken antibiotics in the last 90 days for therapeutic purposes" in Results. | A higher proportion of participants (62.9%) | Page 9 |
| *Awareness & self-medication practices relating to COVID-19 infection - About Table 3: Questions 1 to 4 should be transformed into affirmative sentences assessed by TRUE/FALSE options instead of YES/NO questions. Because they evaluate participants' knowledge, that is the fact and should be followed by TRUE/FALSE options. | Question 1 to 4 have been changed to affirmative sentences as recommended  There is still no approved medication for COVID-19  Self-medication can affect your health adversely  The COVID-19 virus is airborne  The COVID-19 virus spreads via respiratory droplets of infected individuals | Page 11 |
| For example, question 1, "Are you aware there is still no approved medication for COVID-19?" => "There is still no approved medication for COVID-19". | DONE  There is still no approved medication for COVID-19 | Page 11 |
| In this sentence, "Approximately 41% claimed not to be adhering to international guidelines … more than eight times daily.", the authors should clarify which international guidelines were proposed to particpants in their study. | The guideline was the WHO guidelines | Page 11 |
| Factors Associated with Participants' Awareness and self-medication practices - In this sentence, "Those with tertiary educational qualification had higher mean scores relative to participants with primary, high school, and non-formal educational education", the authors have never mentioned how these mean scores were calculated in previous sections. They should specify the method they used for calculating mean scores. | The method has been stated in the data analysis (page 7)  Associations between participants’ demographic characteristics and specific items relating to self-medication practices were analysed using the Chi-square test. A p-value < 0.05 was considered for significant associations. | Page 7 |
| DISCUSSION |  |  |
| The authors should include the limitations of their study. They should have a separate Conclusion section rather than one paragraph to summarize their viewpoints included in the Discussion section. | This study is not without limitations. First, a cross-sectional study was conducted and data were collected at a single time point. Thus, only snapshot information was available and no casual inference could be drawn from this study. Second, the findings are not generalisable to other states in Somalia since only Mogadishu residents were sampled coupled with the relatively small sample size. Third, utilising a quantitative approach and structured questions also has its weaknesses. The technique is prone to response bias and detailed information cannot be gleaned. An in-depth interview and a larger sample size could be considered in future research. | Page 18 |
| MINOR CORRECTIONS |  |  |
| 1/ Results *Prevalence and symptoms for self-medication practices - A higher proportion of participants stated pharmacists' advice (21.4) => A higher proportion of participants stated pharmacists' advice (21.4%) - Meanwhile, common symptoms for which the respondents took medications were fever or chills (15.1%,) headache (14%), while the least reported was loss of smell or taste (1.4%) => while the least reported as a loss of smell or taste (1.4%) *Awareness & self-medication practices relating to COVID-19 infection - Table 4 depicts participants' responses regarding preventive measures and practices against exposure to COVID-19 => Table 3 depicts participants' responses regarding preventive measures and practices against exposure to COVID-19 *Factors Associated with Participants' Awareness and self-medication practices - A higher mean score was obtained by participants aged 25-35 compared other age groups => A higher mean score was obtained by participants aged 25-50 compared to other age groups. - Page 25: Table 6 => Table 5 - About Table 5: The p-value should be written in the same rows with the variables' titles. | All the minor corrections have been effected in the revised manuscript  A higher proportion of participants stated pharmacists' advice (21.4%)  while the least reported as a loss of smell or taste (1.4%)  Table 3 depicts participants' responses regarding preventive measures and practices against exposure to COVID-19  A higher mean score was obtained by participants aged 25-50 compared to other age groups.  Done | Page 9  Page 9  Page 11  Page 11  Page 13 |
|  |  |  |
| REVIEWER 2 |  |  |
| ABSTRACT |  |  |
| Methods: The author should indicate what technique was used for random sampling and statistical analyses in this section. - Results: The authors should provide just a brief balanced summary of what was found in the abstract. | The information has been included  Participants from various disciplines were randomly recruited within the study location and interviewed about their self-medication practices during the COVID-19 pandemic. Descriptive statistics were used to summarise the respondents’ information and responses to the questionnaire items. Associations between participants’ demographic characteristics and specific items relating to self-medication practices were analysed using the Chi-square test. | Page 1 |
| INTRODUCTION |  |  |
| The authors should rewrite their Introduction as they failed to introduce the background of COVID-19 and link to an increased practice of self-medications during the pandemic, as well as to reference the related literature, especially recently published work that showed an increase in self-medication during the pandemic in the target population. - How is self-medication defined? | The introduction has been revised accordingly.  The fear and apprehension following the outbreak of COVID-19 and its subsequent global spread to most continents of the world are palpable in Africa. Factors such as high population density, poorly funded public health sector, and lack of testing facilities in most African countries led to the projections by global [public health experts](https://time.com/5816299/coronavirus-africa-ventilators-doctors/) that the pandemic could be catastrophic in the region [1]. Other critical factors that heightened the fear were inadequate health infrastructure, and a lack of skilled and well-trained human capital to facilitate the collection, processing, and testing of patient samples [2].  From the onset of the pandemic to date, no evidence-based treatments are available for COVID-19 despite the devastation and global spread of the virus. Hence, several countries have resorted to granting authorisation for the use of monoclonal antibody treatments for non-hospitalised adults and children over 12 years old with mild to moderate COVID-19 symptoms [3]. These treatment options are a combination of either casirivimab and imdevimab, or bamlanivimab and etesevimab, recommended especially for those at risk of developing severe COVID-19 or being hospitalised [4,5]. Other less popular options are non-steroidal anti-inflammatory drugs and hydroxychloroquine [4]. Despite the seeming success of these treatments against COVID-19, the World Health Organisation (WHO) declared that clinical trials on these drugs were discontinued due to increased risk of ventricular arrhythmias and death among hospitalised patients [6]. Unfortunately, many African countries have already approved the use of these drugs to treat COVID-19 at the national level as against the WHO directives [6]. The use of these substances without appropriate medical advice as in the case of most African countries is considered self-medication [7].  The WHO defined self-medication as the use of medicinal products, including over the counter (OTC) or non-prescription drugs as well as prescription-only medicines to treat self-diagnosed symptoms or illnesses without obtaining advice from a physician, pharmacist or other healthcare personnel in the diagnosis [8]. It is also the intermittent or continuous use of medication previously prescribed by a physician for chronic or recurring symptoms or diseases. Several studies have reported an increase in the prevalence of self-medication among various populations during the COVID-19 pandemic, including 83% of medical students in Pakistan [9], and 36.3% of healthcare professionals in Nigeria [10], and 34.2% among multidisciplinary participants in Togo [7]. These events have been linked to the widespread psychosis and anxiety induced among populations in sub-Saharan Africa due to the COVID-19 epidemic [11]. Perceivably, the high mortality rates reported in Spain and Italy, as well as the lack of technical resources to address the disease in sub-Saharan Africa [12]. Given the aforementioned situations and the variety of information circulating on social media, numerous substances without the minimum requirements of efficacy and tolerance are taken by individuals either for COVID-19 treatment or prophylaxis [12].  Like most African countries, Somalis practice self-medication due to a lack of regulation and the ease at which drugs can be bought OTC without prescription [13]. This improper usage of drugs for COVID-19 treatment has the potential to complicate case management and induce toxic effects in patients and increase the risk and emergence of drug resistance, which could increase the rate of hospitalisation and severe infection with drug-resistant pathogens [10]. Somalia has been in turmoil for decades and is among the [countries with the weakest health systems in Africa. The country](https://www.ghsindex.org/country/somalia/) is struggling with a new wave of COVID-19 infections with 14,632 confirmed cases and 767 death as of 25th May 2021, and this may likely surge leading to unrecorded and unrecognised deaths [14]. Besides, the reported statistics may not reflect the reality on the ground given that testing for COVID-19 cases has been comparatively limited to major cities, such as Mogadishu, Kismayo, and Marka. Meanwhile, all regions currently facing security challenges are been denied access [14]. Based on the lack of a recognised treatment for COVID-19 and its constant progression, self-medication practices among Somalis need to be evaluated. Given the significance of these issues with the pandemic, this study was conducted to assess the awareness level of COVID-19 and self-medication practices against SARS-CoV-2 viral infection among Somalis. | Page 1 |
| METHODS |  |  |
| The authors should include more information that clarifies and justifies the following: - How was the sample size calculated? And what is the assumed non-response rate? - Which criteria was used to assess the risk of contracting COVID-19 infection in inclusion criteria? - What is the original language of the questionnaire? - What kind of traditional medicine was used? - What are the measurements of variables? - How to assess levels of participants' awareness and self-medication practices | All the information has been included in the revised thesis  The required sample size was calculated using a single proportion formula with a 95% confidence level. We hypothesized that 34.2% of the population will engage in self-medication practice against COVID-19, with a precision error of 5%. This expected prevalence of self-medication practices was chosen based on a study conducted in Togo [7]. The study was used as a reference since it was a cross-sectional design involving participants from sub-Saharan Africa, involving a cross-sectional design. Hence, the estimated minimum sample size was 350. Upon adjusting for a non-response rate of 10%, the sample size was increased to 400 potential participants.  The structured questionnaire was developed in English and no translation process was necessary since the participants use English as a medium of communication.  Self-medication practice was considered the dependent or outcome variable in this study. The variable was measured using a dichotomous item (True/False). Awareness and self-medication practices relating to COVID-19 were also measured using Yes or No responses, however, the items were scored based on participants’ responses (score 1 for a correct answer and 0 for a wrong answer). Thus, the overall score was computed as a continuous outcome. | Page 5 and 6 |
| RESULTS |  |  |
| -What is the response rate? - Table 1: Total number of participants (N=) and data of age are missing - Table 3: what international guideline was used in the study? - The data presented in Table 5 should be expanded and clarified to ensure that readers understand exactly what the researchers studied and demonstrate statistical significance of factors associated with self-medication practice. -To determined factors associated with participants' awareness and self-medication practices, a multivariable analysis should be done. | Response rate was 83.3% (page 8)  The information has been included  According to the WHO guideline  Information regarding the data can be found in the data analysis section  We consider univariate analysis (Chi-square) to be appropriate for the analysis because some of the data used in calculating the awareness scores were missing for a few respondents. | Page 8 |
| DISCUSSION |  |  |
| - The authors failed to identify the limitations of the study (i.e. small sample size) and how to address the bias. | The limitations have been highlighted in a separate section page 18 | page 18 |
| MINOR CORRECTIONS |  |  |
| - The authors need to proofread the manuscript again as there are some grammatical errors and typos. - Table 1, 2, 3: unit of percentage is missing and table 6 was mislabelled. | The article has been proofread accordingly |  |
|  |  |  |
| REVIEWER 3 |  |  |
| Title - Please mention the study design in the title " A cross sectional study ". | The title has been amended  **Self-Medication Practices Against COVID-19 Infection and Awareness among Residents of Mogadishu, Somalia**: **A Cross-sectional Analysis** |  |
| Abstract - It's better to State specific objectives, including the pre-specified hypotheses in separate subheading " Objective" Not in the background. -The Abstract shouldn't exceed 300 words according to PLOS ONE submission guidelines. -Please Summarize the most important results. | The abstract has been revised accordingly  The novel coronavirus disease (COVID-19) pandemic has affected several countries worldwide, resulting in a considerable strain on healthcare systems and increased trend of self-medication practices. This study aims to evaluate the awareness of COVID-19 and the prevalence of self-medication during the pandemic among residents in Mogadishu, Somalia. A cross-sectional study was conducted using a structured and pretested questionnaire between May 2020 and January 2021. Participants from various disciplines were randomly recruited within the study location and interviewed about their self-medication practices during the pandemic. Descriptive statistics were used to summarise the respondents’ information and responses to the questionnaire items. Associations between participants’ demographic characteristics and specific items relating to self-medication practices were analysed using the Chi-square test. A total of 350 residents participated in the study. Approximately 63% of the participants reported having practised COVID-19 related self-medication with the main reasons being pharmacists’ advice (21.4%) and having an old prescription (13.1%), whereas 37.1% did not report their reasons for self-medication. Most participants (60.4%) engaged in self-medication despite not having any symptoms and 62.9% had taken antibiotics in the last three months. Most participants were aware that no medication has been approved for COVID-19 (81.1%), the negative effects of self-medication (66.6%), and the transmission routes of the virus. Meanwhile, more than 40% of the participants have not worn a mask while outside their homes, and do not follow the international COVID-19 guidelines. The most prevalent drug used by participants for self-medication against COVID-19 was paracetamol (81.1%) and antibiotics (78%). The factors associated with awareness of COVID-19 and self-medication practices included age, gender, educational qualification, and occupation. This study revealed considerable high self-medication practices among Mogadishu residents, thus highlighting the need to promote awareness regarding the adverse effects of self-medication and sanitisation guidelines in addressing COVID-19 at the community level. | Page 1 |
| INTRODUCTION |  |  |
| -This sentence " Despite these highlighted challenges highlighted with the United Nations predicting that up to 300,000 deaths" isn't clear, please paraphrase it again. -Please change the citation style into " Vancouver " style according to the Journal guidelines. -Please adjust your citation in this sentence " the ease at which drugs can be bought over the counter without prescription (Reference)." -Please try to provide a brief review of the key literature, the introduction is quite long. | The sentence has been deleted, citation style has been changed to Vancouver style, and all the missing references have been included. The introduction has been shortened | Page 1 |
| METHOD |  |  |
| Please mention how the participants were recruited? -Inclusion and exclusion criteria should be mention in this section with more details. -Please mention the setting and location -How the sample size was calculated? | The information has been provided in the methodology  The required sample size was calculated using a single proportion formula with a 95% confidence level. We hypothesized that 34.2% of the population will engage in self-medication practice against COVID-19, with a precision error of 5%. This expected prevalence of self-medication practices was chosen based on a study conducted in Togo [7]. The study was used as a reference since it was a cross-sectional design involving participants from sub-Saharan Africa, involving a cross-sectional design. Hence, the estimated minimum sample size was 350. Upon adjusting for a non-response rate of 10%, the sample size was increased to 400 potential participants. | Page 4 |
| Data quality control is missed. -Please, mention was the questionnaire in English or not. -How the translation process was done? ( forward or backward…….etc) -were the collected data reviewed and validated before data entry or Not ? and who did the review ? -Has the questionnaire been reformulated after pre-testing or nor?  6. Please, address Any potential source of bias and how did they control the possible effects of confounders? 7. Please mention if your study was funded or not? 8. Strength, limitations and conflict of interests were missed. 9. In table1, why the Age rows are empty? 10. Please change the reference style into " Vancouver" style. | The questionnaire was developed originally in English, so no translation was done. All the other information have been included in the revised manuscript.  No fund was received in conducting this research  Corrected  Done | Page 5 and 6 |
| MINOR CORRECTIONS |  |  |
| Minor Consideration, - Please add line numbering to easily refer to parts that need to be modified. - Describe the abbreviation under your tables. - Avoid unnecessary spaces. - Avoid using capital letters in the middle of the sentences . - Adjust the font of your manuscript according to the journal guidelines. - Please check your manuscript for grammatical mistakes. | Number line has been added and all the suggestions have been considered during the revision  Done |  |
