# Supplementary material for: Self-medication practices against COVID-19 infection and awareness among residents of Mogadishu, Somalia: A cross-sectional analysis
Source: PLoS One. 2023 Jun 28;18(6):e0284854. doi: 10.1371/journal.pone.0284854 (PMC10306195; doi:10.1371/journal.pone.0284854)
Supplement: Peer review history

# Author comment: Author response to previous submission

**DOI:** 10.1371/journal.pone.0284854.r001

12 Mar 2023

Attachment Submitted filename: Response.docx-revised2023_PONE-D-22-11313.docx Click here for additional data file.

# Editor report: Decision Letter 0

**DOI:** 10.1371/journal.pone.0284854.r002

**Authors:** Olushayo Oluseun Olu

11 Apr 2023

Self-Medication Practices Against COVID-19 Infection and Awareness among Residents of Mogadishu, Somalia: A Cross-sectional Analysis

PONE-D-23-04943

Dear Dr. Moussa,

We’re pleased to inform you that your manuscript has been judged scientifically suitable for publication and will be formally accepted for publication once it meets all outstanding technical requirements.

Within one week, you’ll receive an e-mail detailing the required amendments. When these have been addressed, you’ll receive a formal acceptance letter and your manuscript will be scheduled for publication.

An invoice for payment will follow shortly after the formal acceptance. To ensure an efficient process, please log into Editorial Manager at http://www.editorialmanager.com/pone/, click the 'Update My Information' link at the top of the page, and double check that your user information is up-to-date. If you have any billing related questions, please contact our Author Billing department directly at authorbilling@plos.org.

If your institution or institutions have a press office, please notify them about your upcoming paper to help maximize its impact. If they’ll be preparing press materials, please inform our press team as soon as possible -- no later than 48 hours after receiving the formal acceptance. Your manuscript will remain under strict press embargo until 2 pm Eastern Time on the date of publication. For more information, please contact onepress@plos.org.

Kind regards,

Olushayo Oluseun Olu

Academic Editor

PLOS ONE

Journal Requirements:

1. Thank you for stating the following financial disclosure: 

"The authors received no specific for this work "

e) Please provide an amended Funding Statement that declares *all* the funding or sources of support received during this specific study (whether external or internal to your organization) as detailed online in our guide for authors at http://journals.plos.org/plosone/s/submit-now.  

f) Please state what role the funders took in the study.  If any authors received a salary from any of your funders, please state which authors and which funder. If the funders had no role, please state: "The funders had no role in study design, data collection and analysis, decision to publish, or preparation of the manuscript." 

Please send your amended statements by return email; we will change the online submission form on your behalf. 

2. Please include your full ethics statement in the ‘Methods’ section of your manuscript file. In your statement, please include the full name of the IRB or ethics committee who approved or waived your study, as well as whether or not you obtained informed written or verbal consent. If consent was waived for your study, please include this information in your statement as well. 

​

# Editor report: Acceptance letter

**DOI:** 10.1371/journal.pone.0284854.r003

**Authors:** Olushayo Oluseun Olu

15 Jun 2023

PONE-D-23-04943 

Self-Medication Practices Against COVID-19 Infection and Awareness among Residents of Mogadishu, Somalia: A Cross-sectional Analysis 

Dear Dr. Moussa:

I'm pleased to inform you that your manuscript has been deemed suitable for publication in PLOS ONE. Congratulations! Your manuscript is now with our production department. 

If your institution or institutions have a press office, please let them know about your upcoming paper now to help maximize its impact. If they'll be preparing press materials, please inform our press team within the next 48 hours. Your manuscript will remain under strict press embargo until 2 pm Eastern Time on the date of publication. For more information please contact onepress@plos.org.

If we can help with anything else, please email us at plosone@plos.org. 

Thank you for submitting your work to PLOS ONE and supporting open access. 

Kind regards, 

PLOS ONE Editorial Office Staff

on behalf of

Dr. Olushayo Oluseun Olu 

Academic Editor

PLOS ONE
